# Stellate ganglion block efficacy during electrical storm reproduced by hypnotic communication: a case report

**DOI:** 10.1093/ehjcr/ytaf515

**Published:** 2025-10-10

**Authors:** Marco Scaglione, Enrico Guido Spinoni, Veronica Dusi, Domenico Caponi, Gaetano Maria De Ferrari

**Affiliations:** Division of Cardiology, Cardinal Massaia Hospital, Corso Dante 202, Asti 14100, Italy; Division of Cardiology, Cardinal Massaia Hospital, Corso Dante 202, Asti 14100, Italy; Division of Cardiology, Cardiovascular and Thoracic Department, Città della Salute e della Scienza, Corso Mario Achille Dogliotti 41, Turin 10126, Italy; Cardiology, Department of Medical Sciences, University of Turin, Corso Bramante 88/90, Turin 10126, Italy; Division of Cardiology, Cardinal Massaia Hospital, Corso Dante 202, Asti 14100, Italy; Division of Cardiology, Cardiovascular and Thoracic Department, Città della Salute e della Scienza, Corso Mario Achille Dogliotti 41, Turin 10126, Italy; Cardiology, Department of Medical Sciences, University of Turin, Corso Bramante 88/90, Turin 10126, Italy

**Keywords:** Electrical storm, Percutaneous stellate ganglion block, Ventricular arrhythmias, Hypnotic communication, Pharmacological treatment, Surgical gangliectomy, Case report

## Abstract

**Background:**

Percutaneous stellate ganglion block (SGB) is being increasingly used to acutely manage electric storm (ES). No evidence in management of ES with hypnosis is available.

**Case summary:**

We report a case of a 73-year-old man with a severe dilatative cardiomyopathy and ES. Pharmacological therapy was titrated up to the highest tolerated dose (amiodarone, metoprolol, and lidocaine), with ES relapse after 1 week. Percutaneous SGB was effectively performed. Because of neck infection, the infusion catheter was removed and, in order to control arrhythmia relapse before surgical gangliectomy, hypnotic communication, mimicking SGB, was performed. No significant ventricular arrhythmia relapses were recorded for 3 days, when surgical gangliectomy was performed.

**Discussion:**

Hypnotic communication succeeded in reproducing the anti-arrhythmic effects of a percutaneous SGB. Hypnosis, when feasible, might represent a valuable technique for patients with ES.

Learning points

- Electrical storm is a challenging condition that requires a multimodal management: pharmacological therapy, implantable cardioverter–defibrillator reprogramming, sedation, and acute sympathetic tone modulation.
- Hypnotic communication requires a brief training period, it is reproducible, and its applications requires few minutes.
- Hypnosis may act on different mechanisms of electrical storm, reducing anxiety, modulating sympathetic tone, and directly mimicking percutaneous stellate ganglion block.
- Hypnosis does not require drugs; is effective and safe, without side effects; and in the future may play a role in this difficult condition and be part of the physician skills.

## Introduction

Electrical storm (ES) is defined as three or more episodes of sustained ventricular arrhythmias (VA) or of appropriate implantable cardioverter–defibrillator (ICD) interventions during a 24-h period.^1^ Besides pharmacological treatment, percutaneous stellate ganglion block (SGB) is being increasingly used to acutely manage this condition.^2–5^

Lately, hypnotic communication has emerged as a possible treatment strategy for arrythmias.^6–8^ However, clinical experience to control VAs during ES is lacking.

## Summary figure

**Figure:**
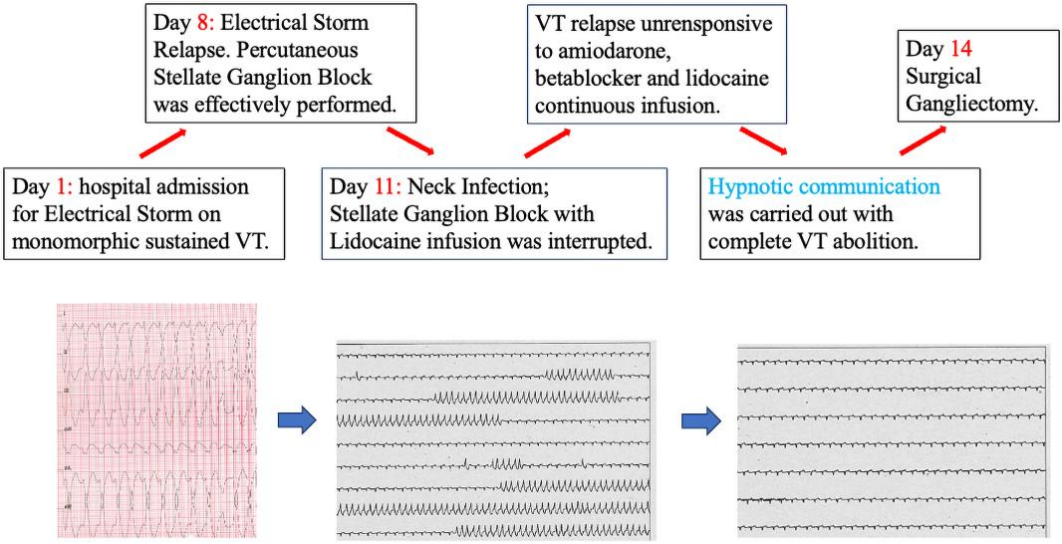


## Case summary

**Figure 1:**
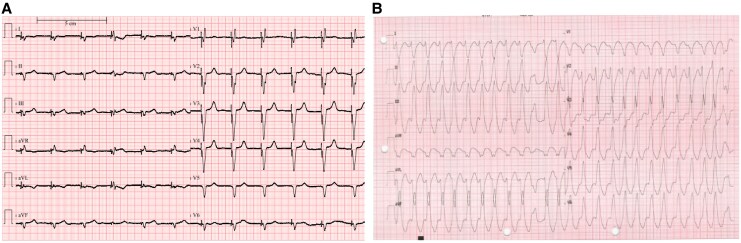
Twelve-lead electrocardiograms: (*A*) biventricular stimulation and (*B*) sustained monomorphic haemodynamically tolerated ventricular tachycardia.

A 73-year-old man presented to our institution for recurrent monomorphic ventricular tachycardias (VTs) at 145 b.p.m. haemodynamically tolerated. The patient was symptomatic for accelerated heartbeat and pre-syncope. Physical examination underlined accelerated heart beats, prosthetic valve click, and no signs of heart failure. Main clinical findings are listed in *Table 1*; notably in the previous month, INR was reported not in range. The arrhythmia caused ICD interventions (ATPs and shock) in several cases. Chronic therapy included amiodarone 200 mg and metoprolol 100 mg b.i.d. (*Figure 1B*). His past medical history included permanent AF; mitral valve replacement with mechanical prosthesis on chronic oral anticoagulation by vitamin K antagonist; percutaneous coronary intervention on the circumflex artery; and heart failure with severe dilatative hypokinetic cardiomyopathy (LVEF 20%) treated with ICD-CRT implantation (patient electrocardiogram (ECG); *Figure 1A*). On admission, i.v. lidocaine was started and oral therapy with amiodarone 200 mg o.d. and metoprolol 100 mg b.i.d. was continued, with partial efficacy. Transthoracic echocardiography showed the presence of LV apical thrombus, which might be related to the previous reported under-range INR. After 1 week, ES relapsed despite titrated medical therapy; consequently, we decided first to try to stabilize the patient by percutaneous left SGB, using the anatomical approach (200 mg lidocaine bolus followed by continuous infusion 2.5 g daily), resulting in complete VA abolition. After 3 days, the infusion catheter was removed due to local neck infection. As the local effect of lidocaine disappeared, non-sustained as well as sustained monomorphic VTs relapsed similarly to the admission time.

**Table 1:** Patient’s main clinical findings upon in-hospital admission

Physical examination	Accelerated heart beats Prosthetic valve click No signs of heart failure
Laboratory exams	Hb 12.1 g/dL WBC 6200 cells/mm³ Creatinine 1.2 mg/dL Na + 137 mEq/L K + 4.1 mEq/L INR 2.1 NT-proBNP 2712 pg/mL HS-TnI 78 ng/L
Transthoracic echocardiography	Presence of mechanical mitral valve prosthesis with regular gradients Global LV hypokinesia EF 20% Regular RV systolic function (TAPSE 20 mm, s’ 12 cm/s, FAC 31%) No pericardial effusion

EF, ejection fraction; FAC, fractional area change; Hb, haemoglobin; HS-TnI, high-sensitivity troponin I; RV, right ventricle; WBC, white blood cells.

In reason of efficacy of the pharmacological SGB, at this point, we decided to continue on this line of therapy referring the patient for surgical gangliectomy.

**Figure 2:**
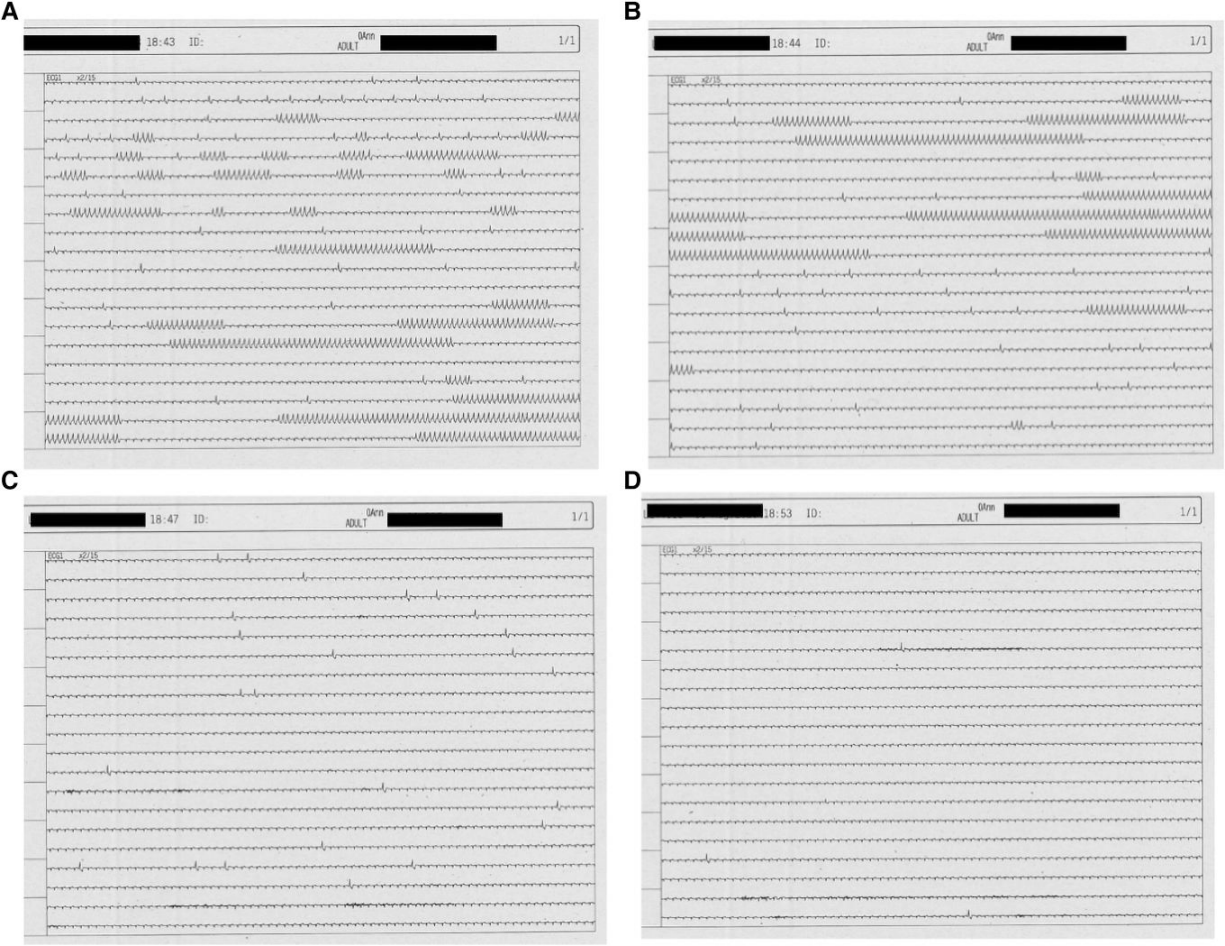
Continuous ECG monitoring showing (*A*) multiple non-sustained ventricular tachycardias before hypnosis, (*B*) ventricular arrhythmia burden reduction after hypnotic communication initiation, and (*C* and *D*) complete absence of ventricular arrhythmias after hypnotic suggestion of stellate ganglion block.

In the meantime, we decided to evaluate the effect of hypnotic communication on VAs performed by an experienced operator aiming to replicate SGB, given the less invasiveness of this strategy and the known reduced efficacy the of the right SGB.^9,10^ Hypnotic communication was carried out while maintaining the previous drug therapy (amiodarone 200 mg o.d., metoprolol 100 mg b.i.d., and continuous lidocaine infusion at the dose of 2 mg/min), unable to modify the arrhythmia burden, which was the same as that at the time of the admission presenting continuous NSVT (*Figure 2A*). Hypnosis was managed using the following workflow, considering as first act the evaluation of the cognitive integrity of the patient crucial to manage the hypnosis, and for this reason, no pharmacological sedation was applied either during the hypnotic phase or in the follow-up up to surgical gangliectomy:

1. Discussion with the patient of the workflow defining the aim (training).
2. Focusing patient’s attention in order to be dissociated from the surroundings.
3. Validation: hypnotic status was assessed by pricking the patient to confirm absence of reactions.
4. Suggestion: the operator guided the patient to reproduce the feeling experienced during the previous pharmacological percutaneous SGB. The operator suggested an image of a control centre positioned in the brain, connected with the heart through an electrical wire passing alongside the neck and having a switch button at the level of the stellate ganglion. To reinforce this concept, the hypnotist touched the neck of the patient at the anatomical site where the lidocaine infusion was previously applied during the SGB. At this time, the operator asked the patient to switch off the stellate ganglion by himself, consecutively interrupting the electrical connection between the brain and the heart.
5. Reinforcement and consolidation: the patient was asked to repeat the block of the stellate ganglion several times.
6. Posthypnotic suggestions (self-hypnosis): the operator suggested the patient to maintain the block until the date of the surgical intervention, and he was trained to reproduce by himself (anchorage) the hypnosis to reinforce the effect during the following days. The arrhythmic drug therapy remained unmodified during the subsequent days, and no pharmacological sedation was applied.

During the hypnosis, the patient’s ECG and vital parameters were continuously monitored, without modification of heart rhythm and blood pressure. After that, the specific suggestions of SGB were given (Workflow 4), and a progressive reduction of VA burden was seen up to a complete abolition that happened in a 5 min time (*Figure 2A–D*).

As usual, in the final part of the hypnosis protocol, the patient was trained to reproduce by himself this hypnotic status and effect (*anchorage*). In such a way, the absence of arrhythmias (no relapse of non-sustained nor sustained VTs) lasted for 3 days when surgery was performed.

The patient underwent surgical gangliectomy after 3 days, with a subsequent reduction of the VT burden without relapse of sustained VTs. He died 7 months later due to refractory heart failure. No significant arrhythmic events were recorded during the follow-up period.

## Discussion

Electric storm is a life-threatening condition and represents a major unfavourable prognostic marker. Its initial management includes anti-arrhythmic drug administration, ICD reprogramming, sedation, and acute sympathetic modulation by SGB. Other advanced anti-arrhythmic strategies after the emergency setting include transcatheter ablation, cardiac sympathetic denervation, and stereotactic ablation.^1,2^

Despite this was a patient with a VT morphology compatible with outflow tract origin suitable for ablation, we first chose a different approach (surgical gangliectomy) by the following considerations:

- ECG morphology is due to the exit site compatible with an RVOT VT; however, this does not exclude a focus/circuit deep in the IV septum requiring a left approach.
- In this case, transcatheter ablation was not contraindicated, according with the current EHRA/ESC guidelines on VT ablation.^1^ However, we considered that in case of need of left approach, the transseptal path was precluded by the presence of mitral mechanical prosthesis and, despite a septal origin, we could not exclude the possibility to interact with the LV thrombus during catheter manipulation.
- Lastly, the poor LVEF of the patient might influence the outcome of the procedure.

Based on these considerations and that, despite the success of percutaneous SGB, the neck infection of the previous infusion site and the greater invasiveness with lower efficacy of right SGB precluded a new percutaneous SGB, we decided to test the effect of hypnosis with specific suggestions to reproduce SGB effect.^9,10^

In this case, the hypnosis was not considered an alternative to ablation, but a bridge to surgical gangliectomy.

Hypnosis is defined as ‘a modified state of consciousness with reduced self-awareness associated with an enhanced capacity for response to suggestion’.^11^ Nowadays, it is well established that hypnosis and traditional analgesia present a synergistic effect; thus, when used, hypnosis reduces the need of analgesics and sedative drugs during invasive procedure.

In the last years, several experiences were reported on hypnosis in the setting of interventional procedures. Our group and other experts have used hypnosis during S-ICD implantation and AF ablation, reducing procedural anxiety and sparing analgesic drugs.^12–15^

Moreover, case reports on arrhythmia management by hypnotic communication have been published, suggesting that hypnosis could be a useful ‘integrative tool’ in the management of supra- and VA.^6–8^ However, limited experiences in this field are available.

Notably, hypnotic communication can induce patient relaxation, anxiety reduction, and, consequently, a favourable autonomic nervous system modulation, with increase of parasympathetic and decrease of sympathetic activity.^16^ These effects may be confirmed by the physical changes frequently observed during hypnosis, such as heart rate modifications, blood pressure decrease, and breathing changes.

In our case, the fast and progressive response with complete abolition of VAs was achieved only after that the suggestion aiming to reproduce the SGB was given by the hypnotist. This effect may be considered a proof that the arrhythmia burden reduction and abolition were not simply induced by the relaxation status given by hypnosis, but by the specific suggestions of SGB. The arrhythmia control in the following 3 days was due to the anchorage of the patient reproducing by himself the effect of hypnosis.

The present case has different limitations. First, the use of hypnotic communication in ES should be tested in a wider population to confirm its efficacy. Moreover, hypnosis can be carried out only in patients without cognitive impairment and without linguistic barrier. Lastly, nowadays, not many hospitals have a certified trained specialist who can perform hypnosis.

Up to date, this report represents the first case of effective treatment of ES by hypnotic communication by reproducing a specific effect mediated by percutaneous SGB.

## Conclusions

Hypnotic communication succeeded in reproducing the anti-arrhythmic effects of a percutaneous SGB. Hypnosis, when feasible, might represent a valuable technique for patients with refractory VAs, in addition to percutaneous SGB before deep sedation or general anaesthesia.

## Lead author biography


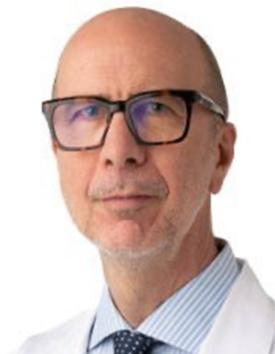


Prof. Marco Scaglione obtained his degree in Medicine and Surgery from the University of Turin in 1989 with honours. Specializing in cardiology, he is currently the Director of the Cardiology Unit at the Cardinal Massaia Hospital in Asti (since 2015) and the Director of the Medicine Department. He is also adjunct professor in Cardiology at University of Verona and consultant for the paediatric cardiology division at the Gaslini Children’s Hospital in Genoa and the Regina Margherita Children’s Hospital in Turin, performing interventional procedures for complex arrhythmias in the paediatric population.

**Consent:** The authors confirm that a written consent for submission and publication of this case report including images and text was obtained from the patient in line with the COPE guidance.

**Funding :** None related to the present manuscript.

## Data Availability

The data that support the findings of this study are available from the corresponding author, upon reasonable request.

## References

[1] Zeppenfeld K, Tfelt-Hansen J, de Riva M, Winkel BG, Behr ER, Blom NA, et al 2022 ESC guidelines for the management of patients with ventricular arrhythmias and the prevention of sudden cardiac death. Eur Heart J 2022;43:3997–4126. 36017572 10.1093/eurheartj/ehac262

[2] Dusi V, Angelini F, Gravinese C, Frea S, Ferrari D. Electrical storm management in structural heart disease. Eur Heart J Suppl 2023;25:C242–C248. 37125278 10.1093/eurheartjsupp/suad048 PMC10132591

[3] Savastano S, Dusi V, Baldi E, Rordorf R, Sanzo A, Camporotondo R, et al Anatomical-based percutaneous left stellate ganglion block in patients with drug-refractory electrical storm and structural heart disease: a single-centre case series. EP Europace 2021;23:581–586. 10.1093/europace/euaa319 33190159

[4] Savastano S, Baldi E, Compagnoni S, Rordorf R, Sanzo A, Gentile FR, et al; STAR study group Electrical storm treatment by percutaneous stellate ganglion block: the STAR study. Eur Heart J 2024;45:823–833.(Erratum in: Eur Heart J. 2024;45(39):4235. doi: 10.1093/eurheartj/ehae631). 38289867 10.1093/eurheartj/ehae021 PMC10919918

[5] Chouairi F, Rajkumar K, Benak A, Qadri Y, Piccini JP, Mathew J, et al A multicenter study of stellate ganglion block as a temporizing treatment for refractory ventricular arrhythmias. JACC Clin Electrophysiol 2024;10:750–758. 38363278 10.1016/j.jacep.2023.12.012

[6] Wain H, Amen DG, Oetgen WJ. Hypnotic intervention in cardiac arrhythmias: advantages, disadvantages, precautions, and theoretical considerations. Am J Clin Hypn 1984;27:70–75. 6209979 10.1080/00029157.1984.10402592

[7] Novoa R, Hammonds T. Clinical hypnosis for reduction of atrial fibrillation after coronary artery bypass graft surgery. Cleve Clin J Med 2008;75:S44–S47. 18540146 10.3949/ccjm.75.suppl_2.s44

[8] Berner A, Tobler C, Reinmann-Assouline M, Degrauwe S, Coen M. Arrhythmia conversion to sinus rhythm during a hypnosis: is hypnosis a normal bystander or a guilty accomplice? Int J Cardiol Heart Vasc 2022;38:100930. 35024427 10.1016/j.ijcha.2021.100930 PMC8724937

[9] Ajijola OA, Lux RL, Khahera A, Kwon O, Aliotta E, Ennis DB, et al Sympathetic modulation of electrical activation in normal and infarcted myocardium: implications for arrhythmogenesis. Am J Physiol Heart Circ Physiol 2017;312:H608–H621. 28087519 10.1152/ajpheart.00575.2016 PMC5402014

[10] Schwartz PJ, Snebold NG, Brown AM. Effects of unilateral cardiac sympathetic denervation on the ventricular fibrillation threshold. Am J Cardiol 1976;37:1034–1040. 1274864 10.1016/0002-9149(76)90420-3

[11] Elkins GR, Barabasz AF, Council JR, Spiegel D. Advancing research and practice: the revised APA division 30 definition of hypnosis. Int J Clin Exp Hypn 2015;63:1–9. 25365125 10.1080/00207144.2014.961870

[12] Scaglione M, Battaglia A, Di Donna P, Peyracchia M, Bolzan B, Mazzucchi P, et al Hypnotic communication for periprocedural analgesia during transcatheter ablation of atrial fibrillation. Int J Cardiol Heart Vasc 2019;24:100405. 31388561 10.1016/j.ijcha.2019.100405 PMC6669807

[13] Scaglione M, Battaglia A, Lamanna A, Cerrato N, Di Donna P, Bertagnin E, et al Adjunctive hypnotic communication for analgosedation in subcutaneous implantable cardioverter defibrillator implantation. A prospective single center pilot study. Int J Cardiol Heart Vasc 2021;35:100839. 34307829 10.1016/j.ijcha.2021.100839 PMC8287220

[14] Scaglione M, Peyracchia M, Battaglia A, Di Donna P, Cerrato N, Lamanna A, et al Subcutaneous implantable cardioverter-defibrillator implantation assisted by hypnotic communication in a patient with Brugada syndrome. HeartRhythm Case Rep 2020;6:198–201. 32322496 10.1016/j.hrcr.2019.12.008 PMC7156977

[15] Garcia R, Bouleti C, Li A, Frasca D, El Harrouchi S, Marechal J, et al Hypnosis versus placebo during atrial flutter ablation: the PAINLESS study: a randomized controlled trial. JACC Clin Electrophysiol 2020;6:1551–1560. 33213815 10.1016/j.jacep.2020.05.028

[16] Ruiz Vargas E, Sörös P, Shoemaker JK, Hachinski V. Human cerebral circuitry related to cardiac control: a neuroimaging meta-analysis. Ann Neurol 2016;79:709–716. 30240034 10.1002/ana.24642

